# Space Flight Diet-Induced Deficiency and Response to Gravity-Free Resistive Exercise

**DOI:** 10.3390/nu12082400

**Published:** 2020-08-11

**Authors:** Shahid Baba, Ted Smith, Jason Hellmann, Aruni Bhatnagar, Kathy Carter, Alexandria Vanhoover, John Caruso

**Affiliations:** 1 Envirome Institute, Department of Medicine, University of Louisville, Louisville, KY 40208, USA; shahid.baba@louisville.edu (S.B.); ted.smith@louisville.edu (T.S.); jason.hellmann@louisville.edu (J.H.); aruni.bhatnagar@louisville.edu (A.B.); 2 Diabetes and Obesity Center, University of Louisville, Louisville, KY 40202, USA; 3 Central State University, Wilberforce, OH 45384, USA; kcarter2@centralstate.edu; 4 Exercise Physiology Program, University of Louisville, Louisville, KY 40208, USA; alexandria.vanhoover@louisville.edu

**Keywords:** immune system, microgravity, ergogenic, nutrition

## Abstract

Immune system dysregulation is among the many adverse effects incurred by astronauts during space flights. Omega-3 fatty acids, β-alanine, and carnosine are among the many nutrients that contribute to immune system health. For space flight, crewmembers are prescribed a diet with a macronutrient composition of 55% carbohydrate, 30% fat, and 15% protein. To quantify omega-3 fatty acid, β-alanine and carnosine intakes from such a diet, and to examine each nutrient’s impact on exercise performance, 21 participants adhered to the aforementioned macronutrient ratio for 14 days which was immediately followed by a workout performed on gravity-independent resistive exercise hardware. Results included daily omega-3 fatty acid intakes below the suggested dietary intake. Daily omega-3 fatty acid, β-alanine and carnosine intakes each correlated with non-significant amounts of variance from the workout’s volume of work. Given the nutritional requirements to maintain immune system function and the demands of in-flight exercise countermeasures for missions of increasingly longer durations current results, in combination with previously published works, imply in-flight supplementation may be a prudent approach to help address the physiological and mental challenges incurred by astronauts on future space flights.

## 1. Introduction

Space flight continues to be one of the most novel and stressful events experienced by humans. The magnitude of physical and mental challenges imposed is only expected to rise as space missions increase in duration and in-flight operational tasks in their complexity. The immune system is at the forefront of the human body’s ability to cope with those challenges [1]. Immune system dysregulation imposed by microgravity contributes to a myriad of in-flight medical conditions that undermine astronaut health. Altered immune cell function from microgravity exposure may elicit chronic unresolved inflammation, which could impact cognitive performance, mood and behavior [1,2,3,4,5]. In addition to space flight as a singular stressor, in microgravity, the immune system is concurrently impacted by in-flight nutrition and exercise, both of which influence the body’s ability to function [6]. Thus in-flight nutrition and exercise protocols are crucial to the success of countermeasure prescriptions administered to astronauts.

There is no singular uniform in-flight diet that is rigidly adhered to during manned space travel. In-flight diets administered to astronauts are varied to avoid boredom and to offer a broader spectrum of nutrients over time. However, since the inception of their manned space flight program, NASA in-flight macronutrient guidelines have remained at 55% carbohydrate, 30% fat, and 15% protein with only limited, and minor, deviations [7,8]. The guidelines were based on the World Health Organization’s energy requirement predictions for moderately active persons, and served astronauts well for space missions, which entailed little to no in-flight physical activity <30 days in duration [7,9,10]. Yet, current and future NASA manned missions include flights for extended stays aboard the International Space Station and to Mars. Such missions will certainly include in-flight exercise countermeasures performed for 1-2 h daily for up to seven days a week [8,11]. While routine, moderate-intensity exercise may best maintain immune system function, microgravity’s deleterious effects on musculoskeletal mass and function necessitate higher intensities, which may in turn compromise the immune system over time [11,12,13]. Under such a scenario, perturbations to in-flight immune system health and function are inevitable and hence nutritional strategies to abate such disturbances are warranted.

Numerous studies documented the impact of omega-3 fatty acids on cardiovascular and immune system health [14,15,16]. Administration of omega-3 fatty acids, especially eicosapentaenoic acid (EPA), alleviates muscle loss associated with cancer cachexia and starvation [15,16]. Clinical trials with humans show supplementation of either EPA or fish oil stabilized weight losses in cachectic pancreatic cancer patients [15,16]. Similarly, omega-3 fatty acid supplementation may inhibit bone losses incurred by astronauts during space flights, and lead to greater inflammation resolution after aerobic exercise [10,11,12,13,14]. Yet the inflammation resolution attributed to greater omega-3 fatty acid intake may also inhibit exercise-induced adaptations; thus, the relative risk-to-benefit ratio of combining exercise with the supplement warrants further inquiry.

In addition to omega-3 fatty acids, the common food constituent carnosine (β-alanine-histidine) improves immune function [17,18]. Carnosine is a dipeptide mostly found in glycolytically active tissues such as the heart, brain, and skeletal muscle. This dipeptide exhibits the abilities to buffer intracellular pH, quell oxygen reactive species, bind metals, form conjugates with lipid peroxidation products, prevent atherosclerotic lesion formation, and improve wound healing [18,19,20,21,22,23]. Evidence from humans show β-alanine supplementation, the rate limiting amino acid precursor for carnosine, sees its levels rise in skeletal muscle and acts as an ergogenic aid to exercise [24,25]. A recent report suggests intracellular carnosine levels increase with intense exercise [25]. Such an adaptation in astronauts may improve their ability to perform in-flight exercise or emergency egress [10,11,25]. Updated dietary requirements for omega-3 fatty acids, β-alanine and carnosine could also mitigate microgravity’s adverse effects and thus they need to be adequately provided through in-flight diets.

During space flight, these nutrients are supplied by a diet with a 55% carbohydrate, 30% fat, and 15% protein macronutrient ratio. Given the physical and cognitive challenges imposed by space flight, which are only expected to increase with future long-term missions, it is crucial that adequate amounts of these nutrients are included in astronaut diets. The current study’s purpose is to quantify omega-3 fatty acid, β-alanine, and carnosine intakes from an isocaloric diet with the same macronutrient ratio as the diet given to astronauts. In addition, the current study will examine correlations between these nutrients and exercise performance done on specialized hardware suggested for in-flight use. It is hypothesized these nutrients obtained from an isocaloric diet with the aforementioned macronutrient ratio will yield significant correlations to the variance in the volume of work performed from workouts.

## 2. Materials and Methods

### 2.1. Design and Study Overview

Prior to admittance in this protocol, which was part of a larger project that compared the in-flight diet to one higher in protein in a randomized within-subjects crossover study, the research work received approval from The University of Louisville’s Institutional Review Board [8]. Healthy physically active (10 men, 11 women) participants were recruited. Each participant’s involvement in the larger project lasted 47 days, which included adherence to the diet with the in-flight macronutrient ratio for 14 days. The diets were separated by a seven-day washout period with no dietary restrictions. The order of intervention diets for each individual was randomized by a coin flip. The project was a single blind study so the principal investigator (JF Caruso) did not know which diet participants were on at the time of testing. Two members of the investigative team were responsible for keeping dietary codes private and making sure participants complied with each diet’s macronutrient requirements. Participants were not allowed to use dietary supplements during their project involvement. Coding was not broken until all data were collected for the larger project [8]. Participants first provided informed written consent, and then filled out a medical questionnaire, which stated they were in good health and free of the following conditions: diabetes, asthma, hypertension, tachycardia, ischemia, arrhythmias, hyperthyroidism, and convulsive disorders. Height and body mass were recorded as they stood barefoot on a stadiometer (Saltner Brecknell; Brooklyn, NY, USA). Body composition was measured by a bioimpedance unit (RJL Systems; Clinton Township, MI, USA) and a metabolic cart (Parvo Medics; Salt Lake City, UT, USA) assessed their energy expenditure rate in a rested state. The NHANES-III equation for the general population was used to measure body composition, while the Parvo Medics TrueOne analyzer quantified energy expenditure via indirect calorimetry. Body composition and energy expenditures values were used to determine each participant’s energy requirements.

### 2.2. Dietary Assessment

Participants kept daily food logs that they submitted to the aforementioned two members of the investigative team. In turn, those members analyzed the logs for their omega-3 fatty acid, β-alanine and carnosine contents. Inspection of food log data showed individual eating patterns deviated little over the 14-day period. Thus, their daily omega-3 fatty acid, β-alanine, and carnosine intakes were good representations of the average values over the 14-day period. Participants were instructed to adhere to the isocaloric diet’s 55% carbohydrate, 30% fat, and 15% protein requirement with no other restrictions on food intake; which meant they could obtain protein from both animal and vegetable sources. This approach was chosen to better mimic the actual in-flight diets astronauts consume. Food logs were examined for omega-3 fatty acid and β-alanine contents with a nutritional analysis program (ESHA Research, Inc.; Salem, OR, USA). Carnosine values were based on estimates that quantified the metabolite’s concentration from muscle biopsies and analyzed by liquid chromatography with tandem mass spectrometry [25].

### 2.3. Measurement of Gravity-Independent Resistive Exercise Capacity

**Figure 1:**
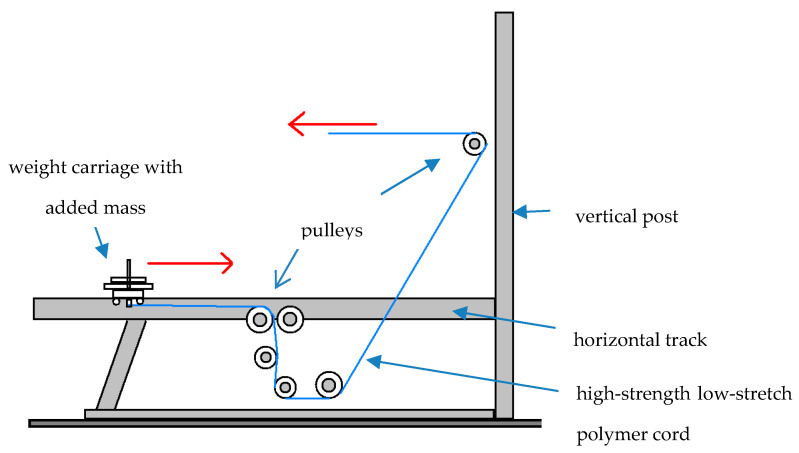
Side view illustration of the inertial exercise trainer (IET; Impulse Technologies; Newnan, GA, USA).

Upon completion of their 14-day diet, each subject immediately performed a workout on a gravity-independent resistive exercise device (Impulse Training Systems; Newnan, GA, USA). Workouts on the device, referred to as an inertial exercise trainer (IET), entailed high-speed high-impact repetitions for four different movements. The IET was chosen based upon its positive impact on the weight-bearing musculoskeleton and potential to serve as in-flight resistive exercise hardware for long-term manned space missions [26,27]. With a series of attachment handles, pulleys, and vertical post settings, the IET permits performance of a variety of movements for the entire body at very high repetition rates and exercise velocities. To achieve this, the external mass added to the IET is light. Since the IET’s weight carriage travels along a 1.9 m track coated with polyurethane, muscle forces as low as ~0.45 N evoke its displacement [28,29]. Thus with a light mass added to the IET, its weight carriage traverses the track at high repetition rates and movement velocities as exercises are performed. Since the carriage oscillates parallel to the Earth’s surface over successive repetitions, such movement is not impacted by gravity; thus, the IET has the potential to serve as in-flight exercise hardware [30]. Figure 1 has a side view illustration of the IET.

**Figure 2:**
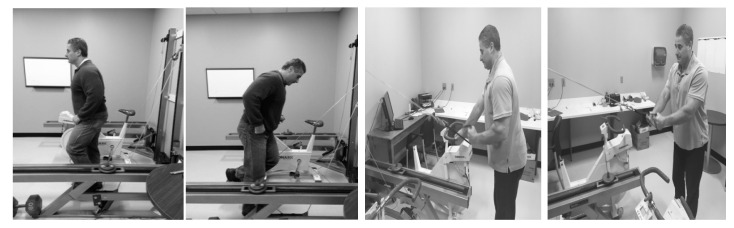
The four movements (standing knee extension, standing hip extension, unilateral row, bilateral pulldown) performed on the IET.

The four movements that comprised workouts were done in the following order: knee extension, hip extension, unilateral row, and pulldown. Participants performed the first three movements with the left side of their bodies as they stood upright, while the pulldown was a bilateral exercise done as they stood upright. With a padded leather cuff as the first movement’s attachment handle, participants performed standing knee extensions, which entailed rapid extension at flexion at their left knee joint as they otherwise remained motionless. The cuff was placed superior to the left ankle’s malleoli for that movement. With the same cuff wrapped around the arch of their left foot, the participant’s standing hip extension movement entailed simultaneous flexion and extension at their left hip and knee joints. For the standing row, participants grabbed an attachment handle with their left hand and pulled it towards them, which entailed concurrent elbow flexion and shoulder hyperextension of their left upper extremity. Antagonistic actions at the aforementioned joints returned the attachment handle back to their original positions at the start of rowing movement. Standing pulldowns required participants to grab attachment handles with both hands and, as they maintained a slight degree of elbow flexion, it involved repetitive shoulder extension and flexion. Figure 2 depicts the four exercise movements.

For each participant, pulley positions were held constant for each of the four movements done on the IET. A 3.4 kg load was added to the carriage, to bring its total resistance to 4.4 kg for each exercise. Workouts occurred under the direction of the principal investigator, who instructed participants to perform each exercise with maximal voluntary effort. Each of the four exercise movements were performed for three one-minute sets separated by 60-s rest periods. With a load cell and position sensor interfaced with the IET, force and carriage displacement were recorded in real time at 1000 Hz. Software (DATAQ Instruments; Akron, OH, USA) housed within a computer that operated the IET was used to calculate the volume of work performed for each exercise movement [30]. The total volume of work was summed for the four exercises and used for statistical analysis.

### 2.4. Statistical Analysis

Our dietary and total volume of work data were first analyzed with Z-scores for the identification of statistical outliers. Our data were then analyzed for their compliance to ANOVA assumptions (normality, independence, homogeneity of variance). Inter-gender comparisons for the participant’s physical characteristics and their omega-3, β-alanine, and carnosine intakes were made with two-tailed t-tests for independent samples. We used Pearson Product Moment Correlation Coefficients to quantify how well each of our dietary measures (average daily omega-3, β-alanine and carnosine intakes) correlated with the variance in our total volume of work data. An α value of 0.05 denoted significance for all our analyses.

## 3. Results

### 3.1. Sample Characteristics

No participants were injured as a result of their project participation. Each participant adhered to the diet, which was affirmed by the two members of the investigative team. Physical characteristics of our participants, as well as their omega-3 fatty acid, β-alanine, and carnosine intakes while they were on their isocaloric 55% carbohydrate, 30% fat, and 15% protein diets, appear in Table 1 and Table 2, respectively. Z-scores identified none of the data as outliers, and each ANOVA assumption was met. The physical characteristics of the participants appear in Table 1. The significant inter-gender differences in Table 1 are like those seen previously [8,28,29].

### 3.2. Omega-3 Fatty Acid, β-Alanine and Carnosine Intakes

Table 2 inter-gender dietary results show a trend (*p* = 0.09) for greater omega-3 fatty acid intakes in the female participants.

### 3.3. Effect of Omega-3 Fatty Acid, β-Alanine and Carnosine Intakes on Resistive Exercise

**Figure 3:**
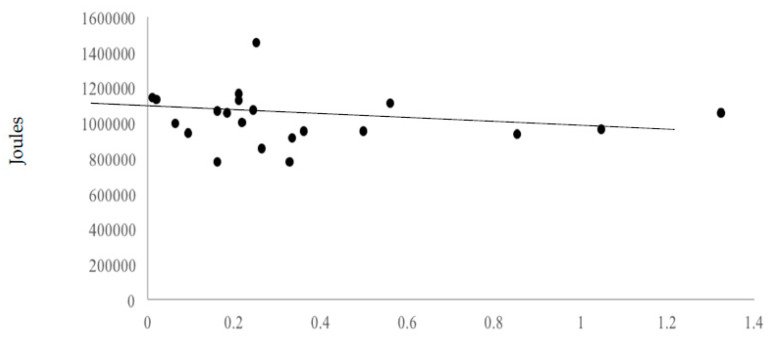
Volume of work performed as a function of average omega-3 fatty acid intakes.

**Figure 4:**
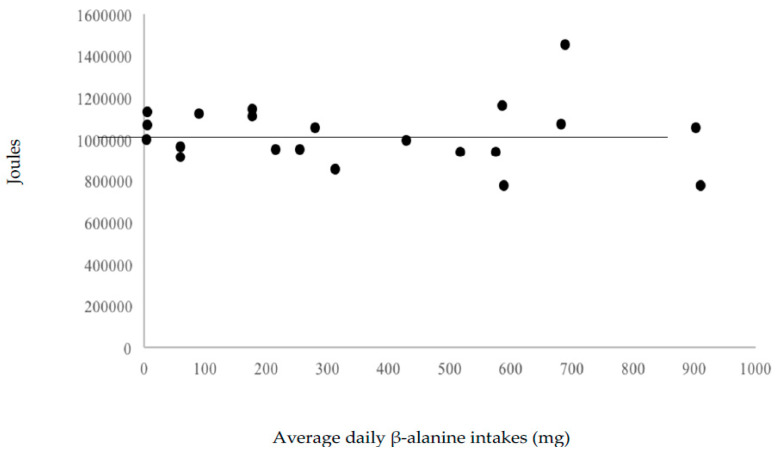
Volume of work performed as a function of average β-alanine intakes.

**Figure 5:**
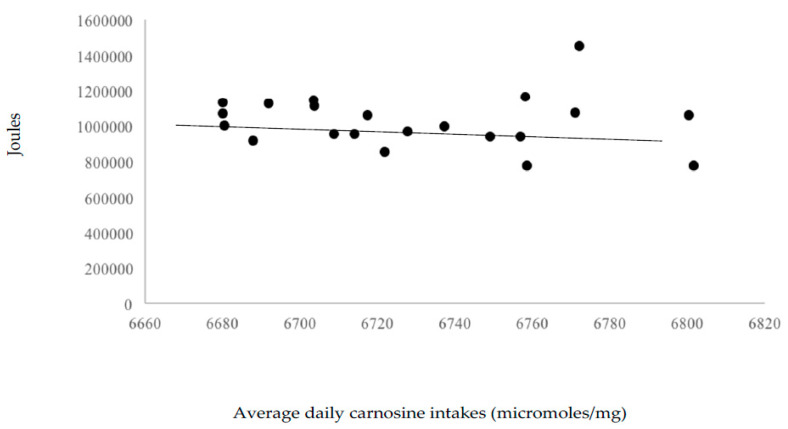
Volume of work performed as a function of average daily carnosine intakes (micromoles/mg).

Neither omega-3 fatty acid (r = −0.11), β-alanine (r = −0.07) and carnosine (r = −0.09) correlated significantly with total work variance. This suggests little effect of these nutrients, at levels provided by the 55% carbohydrate, 30% fat, and 15% protein diet, on workout performance. Figure 3, Figure 4 and Figure 5 display the volume of work performed as a function of omega-3 fatty acid, β-alanine, and carnosine intakes.

## 4. Discussion

Table 2 data variability is a function of participants following their own individual diets, which nonetheless each complied with the 55% carbohydrate, 30% fat, and 15% protein requirement. We must reject the current study’s hypothesis that daily omega-3 fatty acid, β-alanine, and carnosine intakes would correlate with significant amounts of total work variance. Current results, in comparison to positive outcomes seen in related studies [6,13,14,15,16,17,18,19,25], were unexpected. Throughout the 14-day dietary period, the current study participants were not administered a structured exercise intervention or ingested supplements for the three nutrients under investigation, either of which would have increased the concentrations of those same nutrients [6,13,14,15,16,17,18,19,25]. Yet, in relation to the studies that saw positive outcomes [6,13,14,15,16,17,18,19,25], the inclusion of current study data suggests that the totality of evidence on this topic makes a strong case for dietary supplementation, and the 55% carbohydrate, 30% fat, and 15% protein macronutrient ratio while on an isocaloric diet may not offer enough omega-3 fatty acid, β-alanine, and carnosine for long-term manned space missions.

The nutritional demands of space flight are considerable. Providing astronauts with sufficient nutrients during space travel is made more difficult by in-flight appetite suppression and inadequate food intake that inevitably impairs immune system function [1,31]. Foods for consumption during space travel were initially developed by The US Air Force School of Aerospace Medicine in conjunction with The US Army Natick Laboratories and NASA [7]. Macronutrient guidelines were originally devised around that same time period. Currently there are no in-flight guidelines for omega-3 fatty acid, β-alanine and carnosine intakes, which play an essential role in healthy immune function and exercise performance. Likewise, there are no recommended daily allowance (RDA) requirements for these nutrients. Suggested dietary intakes for omega-3 fatty acids are 1.6 and 1.1 g day^−1^ for men and women respectively [32,33]. We found daily intakes of omega-3 fatty acids for current male and female participants as they consumed the 55% carbohydrate, 30% fat, and 15% protein diet were far less than the suggested intake levels. Although speculative, particularly without RDA values, the average intake of carnosine is ~1.3 g day^−1^. Furthermore omega-3 fatty acid, β-alanine, and carnosine intakes from the 55% carbohydrate, 30% fat, and 15% protein macronutrient ratio failed to correlate with significant amounts of total work variance. Collectively our results suggest ergogenic, health, and immune system benefits from higher omega-3 fatty acid, β-alanine, and carnosine intakes are lacking in the diet examined in this study.

Immune system function is markedly translational, with the totality of changes produced by space flight unlike those seen elsewhere. For instance with space flight certain features of adaptive immunity are dysregulated, while some characteristics of innate immunity are heightened. Immunity is impacted by diet and exercise [1,11]. Routine moderate-intensity exercise lowers the risk of chronic disease formation likely through decreases in chronic inflammation and inflammatory mediator production [12]. Exercise recently enhanced the resolution of acute inflammation in mice by augmenting actions of the anti-inflammatory, proresolving lipid resolvin D1, which in turn enhances macrophage phagocytosis [13]. Epinephrine levels, which rise during exercise, stimulate resolvin D1 and macrophage phagocytosis through an α1 adrenergic receptor-dependent mechanism, and thereby enhances resolution of acute inflammation [13]. Resolvin D1 is produced from conversion of the omega-3 acid docosahexaenoic acid, and therefore limited availability of omega-3 fatty acids may predispose the development of chronic inflammation. The lower kilocalorie intakes seen in-flight are associated with inflammation, DNA damage, and oxidative stress [1]. In contrast, omega-3 fatty acids assist the immune system against oxidative damage and radiation-related risks incurred from space flights [31].

Nuclear transcription factor NF-_K_B, which activates numerous genes such as those that evoke sarcopenia and bone demineralization, increases ~500% from short-term space flights and stays elevated for 14 days post-flight [14]. Yet, EPA attenuates that adverse change from space flight and its analogs. Specifically EPA inhibits the signaling of NF-_K_B that leads to inflammation and the subsequent activation of the ubiquitin proteolytic pathway as well as heightened osteoclast activity [14]. Thus immunologic countermeasures now under consideration include omega-3 fatty acid supplementation for long-term flights [1].

Positive effects on bone, muscle, and the inflammatory cytokine TNFα also occur with higher omega-3 fatty acid intakes [1]. Higher dietary intakes of fatty fish tended to preserve whole-body bone mineral contents to a greater extent than diets lower in fish. In a study of omega-3 fatty acid intakes on bone mineral content changes in older adults, participants were divided into high (>1.27 g day^−1^) and low (<1.27 g day^−1^) consumption groups, with better results seen in the former group [34]. Using the classifications from the aforementioned study, current omega-3 fatty acid intakes are far less than the high consumption group despite adhering to the macronutrient guideline prescribed to astronauts [34]. Current results show adherence to that macronutrient guideline while on an isocaloric diet led to an insignificant correlation between omega-3 fatty acid consumption and total work volume and, based on prior results, may not adequately address adverse changes to musculoskeletal and immune system physiology incurred from space flight [1,11,14,31,34].

In addition to omega-3 fatty acids, numerous reports show carnosine, a food constituent present in red meat, or its precursor β-alanine, improves exercise performance and immune system function [24,25]. Carnosine homeostasis within glycolytically active tissues is governed by multiple factors such as the rate of β-alanine uptake into cells, synthesis by carnosine synthase, hydrolysis by carnosinase 1 and 2, expression of transporters PEPT1 and PEPTs, as well as hepatic synthesis and transport [35,36,37,38,39,40,41]. Since β-alanine is the rate limiting amino acid for carnosine synthesis, it is widely used by athletes as an ergogenic aid, particularly if their diet is unable (i.e., vegetarians) to provide adequate amounts of the amino acid.

A meta-analysis of β-alanine supplementation showed a median intake of 179 per study elicited an average ergogenic benefit of less than 3% [37]. The benefits of β-alanine supplementation as an ergogenic aid were greatest for exercise bouts that lasted between 60–240 s [24]. A recent study assessed oral β-alanine (3 g day^−1^) in untrained participants to assess the singular effect of the supplement devoid of any synergistic effects provided by concurrent training [24]. The study had a within-subjects design whereby participants ingested the supplement or placebo for 30 continuous days, and followed each 30-day period with a seated leg press workout [24]. Versus the placebo condition, results showed the β-alanine treatment had significantly greater average power outputs, and higher blood lactate values at zero minutes post-exercise. It was concluded β-alanine supplementation enabled higher power outputs despite increased acidosis due to a heightened buffer capacity, presumably due to greater intracellular carnosine [24]. Because carnosine exhibits a multifarious biochemical profile, the mechanisms by which increased carnosine synthesis results from β-alanine are unclear.

Recently the effects of exercise were compared, with and without β-alanine supplementation (6.4 g day^−1^) on changes in intracellular carnosine levels and exercise performance [25]. Each group performed three weeks of endurance training, followed by six weeks of high-intensity interval training. It was found that placebo-dosed participants had a 24% increase in their muscle carnosine content after three weeks of endurance training [25]. However after six weeks of high-intensity training, their muscle carnosine contents declined. Yet, the group who concurrently ingested β-alanine had a 51% increase in muscle carnosine after three weeks of endurance training, and after the six weeks of high-intensity training there was a 127% increase in muscle carnosine [25]. It was also found the lipid peroxidation products generated during high intensity training were conjugated with carnosine, which suggested the removal of these toxic products could improve immune function [25]. Numerous reports showed carnosine supplementation improves immune function, enhances wound healing and diminishes atherosclerotic lesion formation by removal of lipid peroxidation products [18,19,39]. Hence, β-alanine supplementation may be an effective strategy to limit aldehyde-induced toxicity in skeletal muscle during strenuous activity, such as that which could be produced by future in-flight exercise workouts during long-term space missions [25]. In the current study, when we compared the intake of β-alanine and rate of carnosine synthesis from the space flight diet, it was found the recommended levels of carnosine necessary to improve immune health and exercise performance were too low. Humans consume on average 280 g of meat/day which provides 1.3 g day^−1^ of carnosine. In our analysis of the 55% carbohydrate, 30% fat and 15% protein diet data, the β-alanine intake and the amount of the carnosine that could be synthesized was ~15–16 fold less compared to average daily dietary intakes. Hence the macronutrient guidelines prescribed to astronauts are in stark contrast to results from studies that show heightened intracellular carnosine levels from β-alanine supplementation and improvements in chronic exercise [24,25].

Current results, whereby participants merely adhered to their normal physical activity patterns as they complied with in-flight macronutrient intake guidelines, suggest the diet lacks adequate omega−3 fatty acid, β-alanine, and carnosine intakes. While an RDA for β-alanine and carnosine does not exist, current results show omega-3 fatty acid intakes were far less than those suggested as adequate, or deemed as high, by prior research [32,33,34]. Similarly, the intake of carnosine was also significantly lower compared to average human dietary intakes. Hence, the current omega-3 fatty acid, β-alanine and carnosine intakes from the 55% carbohydrate, 30% fat, and 15% protein macronutrient ratio is insufficient to improve immune health. In contrast, diets rich in these nutrients, or through a combination of supplementation and/or exercise, improved immune system and musculoskeletal health [1,6,12,13,14,20,21,22,23,24,36,37]. Given (1): the nutritional requirements to maintain immune system function and the demands of in-flight exercise for missions of increasingly longer durations [1,2,7,9,10,11,14,31], and (2): the beneficial effects of adequate nutrition, with or without concurrent exercise, on immune system and musculoskeletal health [1,2,7,9,10,14,31], current results, in combination with previously published works, imply in-flight supplementation of omega-3 fatty acids, β-alanine and carnosine may be a prudent countermeasure to help abate the physiological and mental challenges incurred by astronauts on future long-term space flights.

## 5. Implications/Future Recommendations

It is important to acknowledge the current study limitations of no microgravity simulation, and the participants did not consume foods specifically prepared for space flight, but rather simply adhered to the macronutrient guidelines. Meeting nutrient demands of astronauts as they reside within a closed food system often leads to suboptimal intakes of nutrients like those examined in the current study [1,7,11]. Yet, current results were not obtained from subjects within a closed food system, but merely ate as they wished as long as they complied with the 55% carbohydrate, 30% fat, and 15% protein macronutrient ratio, which still led to suboptimal intakes of omega-3 fatty acids, β-alanine, and carnosine. In-flight supplementation would likely provide nutrients to astronauts with fewer storage, payload shipment (i.e., reduced mass), and safety (freshness expiration) concerns than foods currently delivered to manned space missions. These nutrients, when combined with exercise, generally produce greater physiological and ergogenic effects; this would certainly improve the quality of in-flight exercise countermeasures to abate the adverse physiological deconditioning and immune system dysregulation seen in astronauts [1,6,7,11,12,13,14,21,22,23,25,36]. Based upon current results and the body of research available, future studies should examine omega-3 fatty acid, β-alanine and carnosine supplementation in conjunction with chronic exercise training in space flight and its analogs (i.e., bed rest) with an emphasis on musculoskeletal and immune system outcomes.

## Figures and Tables

**Table 1:** Physical characteristics (mean ± sem) of our participants.

	Women (n = 11)	Men (n = 10)	Total (n = 21)
Height (cm)	162.1 ± 2.0	178.6 ± 2.6 *	170.2 ± 1.4
Body mass (kg)	68.5 ± 2.0	80.2 ± 2.8 *	74.1 ± 1.1
Body fat (%)	31.2 ± 2.2 ^#^	14.7 ± 0.6	22.5 ± 2.2
Fat free mass (kg)	48.6 ± 1.2	69.4 ± 3.0 *	58.5 ± 3.1
Body Mass Index (kg/m^2^)	26.3 ± 0.9	25.3 ± 1.0	25.6 ± 0.4

*: significantly greater than corresponding female value. #: significantly greater than corresponding male value.

**Table 2:** Participant’s omega-3, β-alanine and carnosine daily intakes (mean ± sem).

	Women (n = 11)	Men (n = 10)	Total (n = 21)
omega-3 fatty acids (g)	0.47 ± 0.13	0.22 ± 0.05	0.35 ± 0.07
β-alanine (mg)	369.5 ± 80.3	373.3 ± 101.8	371.3 ± 62.5
carnosine (micromoles/mg)	6729.3 ± 10.7	6729.9 ± 13.6	6729.6 ± 8.3
kilocalories	2204 ± 181	2800 ± 226	2484 ± 157
CHO (g/55% of energy intake)	303 ± 22	385 ± 27	335 ± 21
Fat (g/30% of energy intake)	74 ± 9	93 ± 10	82 ± 5.4
Protein (g/15% of energy intake)	83 ± 8	105 ± 9	91 ± 7
